# LiNi_1/3_Mn_1/3_Co_1/3_O_2_ nanoparticles produced by flame spray pyrolysis with crystallinity characteristics similar to commercial NMC particles†

**DOI:** 10.1039/d5ra02976g

**Published:** 2025-08-07

**Authors:** Xueyan Zhao, Peter Benedek, Konstantin M. Engel, Florian M. Schenk, Jasper Clarysse, Ramesh Shunmugasundaram, Annelies Landuyt, Christoph R. Müller, Wendelin J. Stark, Vanessa Wood

**Affiliations:** a Materials and Device Engineering Group, Institute for Electronics, Department of Information Technology and Electrical Engineering, ETH Zurich Gloriastrasse 35 8092 Zurich Switzerland vwood@ethz.ch; b Department of Chemical Engineering, Stanford University Stanford CA 94305 USA; c Functional Materials Laboratory, Institute of Chemical and Bioengineering, Department of Chemistry and Applied Biosciences, ETH Zurich Vladimir-Prelog-Weg 1 Zurich 8093 Switzerland; d Chemistry and Materials Design Group, Institute for Electronics, Department of Information Technology and Electrical Engineering, ETH Zurich Gloriastrasse 35 8092 Zurich Switzerland; e Laboratory of Energy Science and Engineering, Institute of Energy Technology, Department of Mechanical and Process Engineering, ETH Zurich Leonhardstrasse 21 Zurich 8092 Switzerland

## Abstract

To achieve higher energy densities in lithium-ion batteries, improvements in the battery cathode performance are crucial. As cathode materials, nickel-rich layered transition metal oxides play an important role in the market. However, they suffer from surface degradation which contributes to the capacity fade. Using nanoparticles, which offer a large surface to volume ratio, these surface degradation reactions can be better understood. But to do so, nanoparticles with properties similar to those of primary particles in commercial NMC are necessary. In this work, we present the synthesis of sub-100 nm of LiNi_1/3_Mn_1/3_Co_1/3_O_2_ (NMC111) nanoparticles through a flame spray pyrolysis and post-calcination. We study the phase purity and electrochemical performance of the NMC111 nanoparticles as a function of the calcination temperature and demonstrate that optimizing the calcination temperature enables us to achieve a pure layered phase and electrochemical performance on par with commercial NMC111 particles. Mild acid treatment can be used to remove surface impurities that develop with air exposure and improve the long-term stability.

## Abstract 2

NMC111 nanoparticles prepared from flame spray pyrolysis, calcined at 600–800 °C, match commercial structure and performance. Their high surface-to-volume ratio makes them an ideal platform for probing NMC surface degradation.

## Graphical abstract

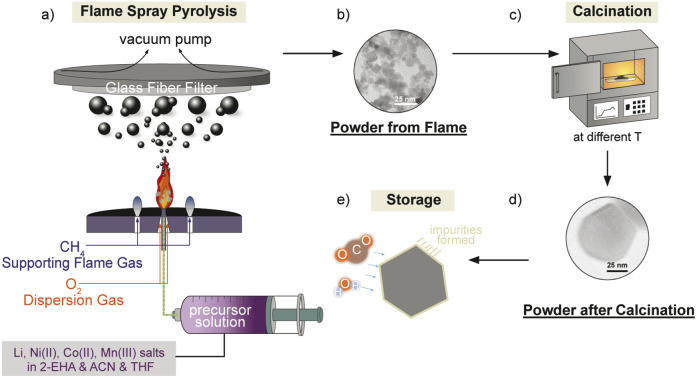

## Introduction

LiNi_1−*x*−*y*_Mn_*x*_Co_*y*_O_2_ (NMC) layered oxide cathode materials have been widely used in Li-ion batteries for electric vehicles due to their high theoretical capacity and operating voltage.^1^ The surface of NMC family cathode materials is vulnerable to side reactions,^2^ including the reactions with electrolyte,^3^ transition metal dissolution,^4^ structure degradation and reconstruction,^5,6^ oxygen release,^7^ and cracking.^8^ Commercial NMC powders are typically prepared *via* co–precipitation reactions,^9^ resulting in spherical micrometer-sized secondary particles, composed of sub-micron or nano-sized primary particles.^10^ This shape minimizes the surface to volume ratio, alleviates the side reactions on the electrolyte–particle interface, and improves the cycling stability. However, the secondary particles can exhibit particle cracking,^11^ due to the isolated active material particles and increased surface area,^12,13^ eventually resulting in capacity loss.

Recently, single-crystal NMC materials, composed of monolithic grains on the order of several microns, have drawn intense research interest.^14–17^ Their lack of grain boundaries enhances the mechanical integrity, effectively suppressing the formation of intergranular cracks.^18,19^ Despite these advantages, the single-crystal structure can develop intragranular fracture during the calendaring process,^20,21^ and they remain susceptible to the intrinsic NMC surface issues such as transition metal dissolution^22^ and surface reconstruction.^23^

When studying the performance of state-of-the-art micron-sized NMC particles by commonly used bulk characterization techniques (electrochemical measurements, XRD, Raman, FTIR, XANES, *etc.*), it is difficult to distinguish surface reactions from reactions due to chemomechanical stresses occurring within the bulk of the secondary particles, making it challenging to propose effective strategies for improvement. While XPS^24,25^ and STEM-EELS are surface-sensitive, they are more resource-intensive and often provide localized information, potentially not representative of the entire active materials. In contrast, the use of nano-sized particles in this study provides an ideal model for studying surface properties and degradation investigations. They inherently represent the fundamental building blocks of secondary particles and single crystal NMC, but eliminate the complexities of intergranuar fracturing within secondary structures. Furthermore, thanks to their high surface-to-volume ratio, interface-related changes are amplified, enabling easier detection by a wider range of characterization techniques, including bulk-averaging methods.

Many efforts have been made to synthesize NMC nanoparticles. State-of-the-art NMC nanoparticle syntheses include hydrothermal,^26,27^ sol–gel,^28,29^ solid-state,^30,31^ combustion,^32,33^ and electrospinning^34^ methods. Flame spray pyrolysis (FSP) is a versatile technique for producing nanostructured metal oxide powders *via* combustion of appropriate precursor droplets.^35^ It provides the benefits of low cost, scalability, and the ability to control both particle size and crystallinity.^36^ While several studies have fabricated NMC particles using FSP^33,37,38^ and have examined aspects of their electrochemical properties, a comprehensive investigation of how the particle size and crystal structure evolves with the post-calcination temperature, and how these changes correlate to the electrochemical properties, has not yet been reported.

In this study, we discuss the synthesis of LiNi_1/3_Mn_1/3_Co_1/3_O_2_ (NMC111) nanoparticles of different sizes *via* FSP and the effects of post-calcination. We study the crystal structure of the particles as a function of the calcination temperature and explore their size-dependent electrochemical properties. We then provide a practical approach to removing surface impurities, which have been observed in previous studies as a result of air exposure.^39,40^

## Experimental

### Synthesis of materials

**Fig. 1:**
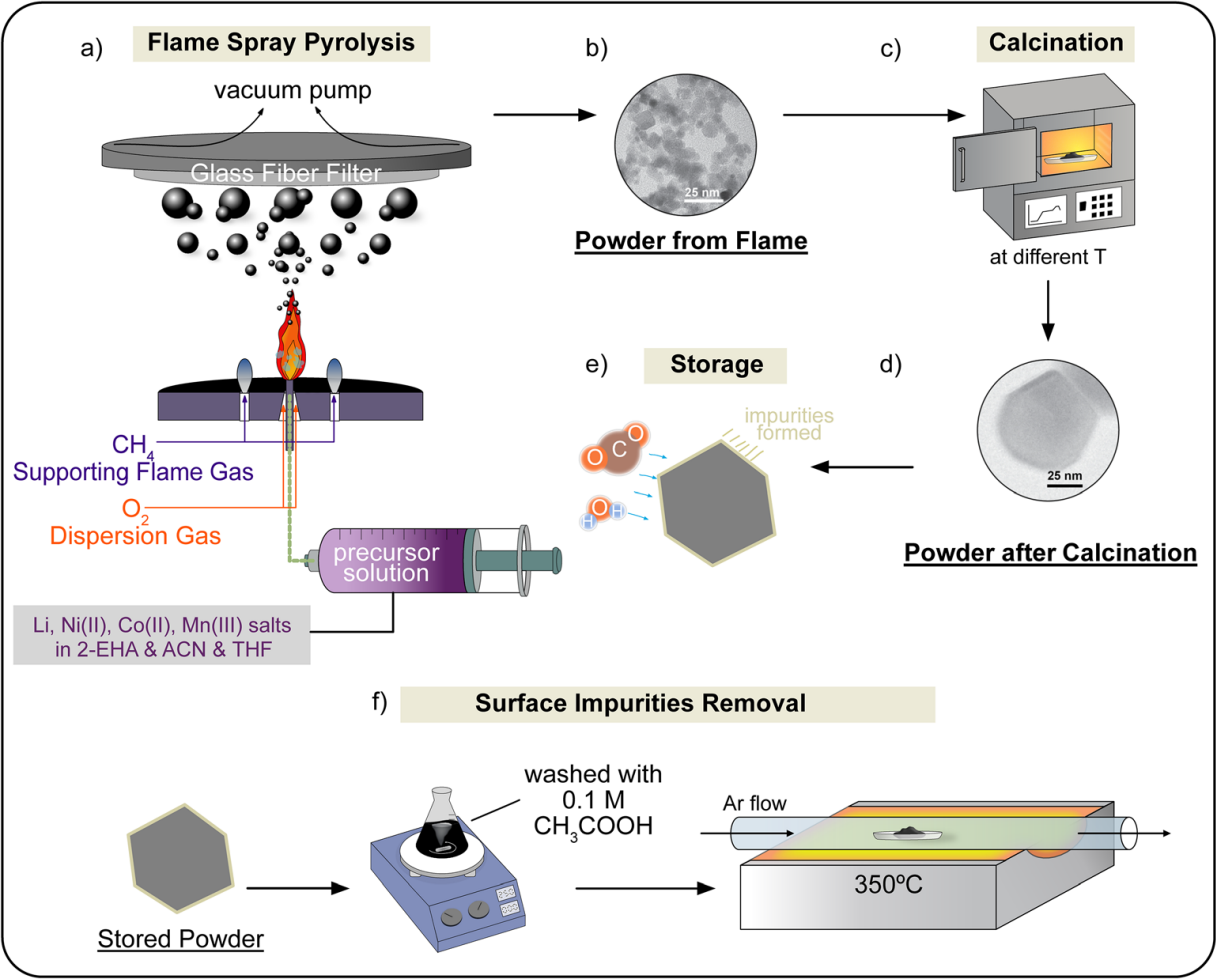
Depiction of the LiNi_1/3_Mn_1/3_Co_1/3_O_2_ (NMC) nanoparticle synthesis. Flame spray pyrolysis yields nanopowder (a and b), which is then calcined (c and d) to achieve a high-performance NMC nanopowder. The calcined powder inevitably encounters air exposure, which degrades the surface (e). Surface impurities can be removed *via* acid wash and subsequent drying (f).

LiNi_1/3_Mn_1/3_Co_1/3_O_2_ (NMC111) nanoparticles were synthesized by flame-spray-pyrolysis (FSP),^35,41–43^ followed by post-calcination at different temperatures (see Fig. 1a–d). To prepare the precursor solution for FSP, stoichiometric amounts of lithium acetylacetonate (97%, Sigma-Aldrich), nickel(ii) acetylacetonate (95%, Sigma-Aldrich), manganese(iii) acetylacetonate (technical grade, Sigma-Aldrich), and cobalt(ii) 2-ethylhexanoate (12 wt% Co, in mineral spirits, ABCR) were dissolved in a 40/40/20 wt% solvent mixture of acetonitrile (99.8%, Sigma-Aldrich), 2-ethylhexanoic acid (99%, Acros Organics) and tetrahydrofuran (for chromatography, Merck). To compensate for the loss of lithium in the high-temperature process, 5 at% excess of lithium was added to the precursor solution. The total metal concentration of the precursor solution was 0.6 mol kg^−1^. The feeding rate of the precursor solution was 5 mL min^−1^, and the flow rate of O_2_ for dispersion was 5 L min^−1^ at a pressure of 1.5 bar. The product was collected using GF/6 glass fibre filters. The resulting powder from pyrolysis was then calcined in air at temperatures ranging from 600 °C to 800 °C for 8 h, yielding the final NMC111 nanoparticles.

To remove the surface impurities, some NMC particles were etched for 5 min in a solution of 0.1 mol L^−1^ acetic acid in distilled water (see Fig. 1e and f). The cleaned NMC powder was then separated from the solution *via* centrifugation and dried under vacuum at 80 °C for 8 h. The dry powder was heated at 350 °C under an argon atmosphere for 2 h to remove volatile organic compounds.

### Materials characterization

The structure of the synthesized NMC111 nanoparticles was characterized by X-ray diffraction (XRD), measuring at room temperature in a 2*ϑ* range of 10° to 70° with a step size of 0.01°, using a Rigaku SmartLab 9 kW system equipped with a Cu Kα radiation source in parallel-beam geometry. The acquired XRD patterns were fitted *via* Rietveld refinement using GSAS II,^44^ with background treatment applied using a polynomial fitting function. The XRD patterns of the reference structure were obtained from the ICSD database.^45^ Fourier-transform infrared (FTIR) spectra were collected on a Bruker Vertex 70 with the KBr pellet method. The composition of as-synthesized nanoparticles was analyzed by inductively coupled plasma mass spectrometry (ICP-MS, Nexlon 350 D, PerkinElmer). N_2_ physisorption experiments were performed on an Anton Paar Nova 800 analyzer. The surface area of the materials was determined by fitting the adsorption isotherm with a density functional theory (DFT) model for N_2_ physisorption in cylindrical pores of nonpolar materials. Transmission electron microscopy (TEM) imaging was carried out on a Hitachi HT7700 microscope. Secondary electron images were acquired on a Hitachi SU-8200 scanning electron microscope (SEM). TGA measurements were carried out on a Mettler-Toledo TGA/DSC 3+ under dry N_2_ flow (150 mL min^−1^) with a heating rate of 10 °C min^−1^.

### Electrochemical characterization

A slurry of 70 wt% synthesized NMC111 nanoparticles, 20 wt% carbon black (Super C65, MSE Supplies), and 10 wt% polyvinylidene fluoride (PVDF, Kynar HSV900) mixed and dispersed in *N*-methyl-2-pyrrolidone (NMP, Sigma), was cast onto an aluminum foil. As a reference, a slurry of commercial NMC111 (MTI Corporation, non-coated) was also prepared in the same way. Slurries were dried at 120 °C under vacuum. After drying, electrodes were calendered before use.

Coin-type cells were assembled in an Ar-filled glovebox with an NMC111 working electrode, a glass fiber separator, a lithium metal counter electrode, and a 1 M electrolyte solution of LiPF_6_ in 1 : 1 (w/w) ethylene carbonate and ethyl methyl carbonate (LP50, Gotion). Galvanostatic cycling tests were performed at 25 °C on a biologic MPG2 potentiostat. The cells were charged and discharged between 2.8 V and 4.3 V. The long-term cycling test consisted of four formation cycles at a C-rate of C/10 and a hundred cycles at C/3. Electrochemical impedance spectra (EIS) were recorded from 100 kHz to 1 mHz (8 points per decade, 5 mV amplitude) after one C/10 formation cycle. The Galvanostatic Intermittent Titration Technique (GITT) was carried out using 15 min C/20 current pulses followed by 2 h relaxation interval, after a single C/10 formation cycle.

## Results and discussion

### Physical characterization

**Fig. 2:**
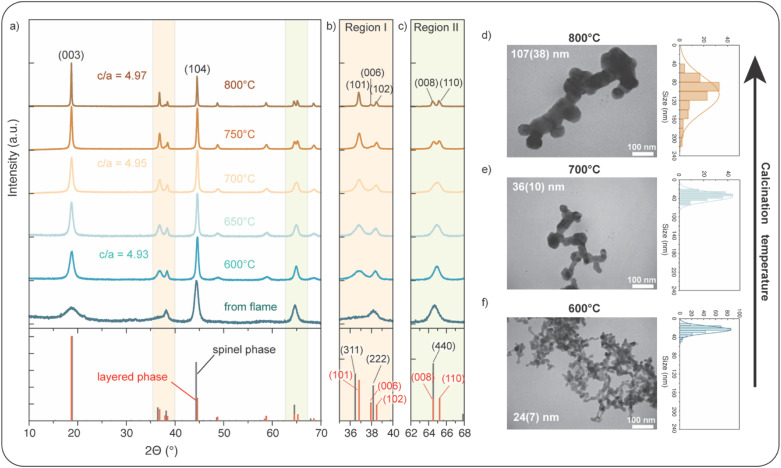
Structure and morphology of NMC111 nanoparticles. (a) XRD patterns of the from flame and after calcining at different temperatures from 600 to 800 °C. XRD patterns of LiNi_0.5_Mn_1.5_O_4_ (spinel phase, *Fd*3̄*m*, ICSD-8408 (ref. 46)) and LiNi_0.33_Mn_0.33_Co_0.33_O_2_ (layered phase, *R*3̄*m*, ICSD-171750 (ref. 47)) are shown as a reference. (b) Zoomed-in view of XRD Region I (35–40° 2*θ*), highlighting evolution of (006)/(102) peaks. (c) Zoomed-in view of XRD Region II (62–68° 2*θ*), showing changes in (008)/(110) peaks. (d–f) TEM images of NMC111 nanoparticles calcined at 800, 700, and 600 °C, and their corresponding size distribution histograms.

Table 1 presents the physical characterization of the as-synthesized powder collected from FSP and after calcining at various temperatures from 600 °C to 800 °C. During the calcination process, the initial grains grow, fuse, and neck together, as shown in Fig. 2d–f, S1 and S3a.† We find that the 1 : 1 : 1 ratio of transition metals remains constant; however, with increasing temperature, the grain size increases from 24 ± 7 nm to 107 ± 38 nm, and the specific surface area of the particles decreases from 44 m^2^ g^−1^ to 8.9 m^2^ g^−1^.

**Table 1:** Physical properties of the synthesized NMC111 nanoparticles calcined at various temperatures: grain size measured using TEM images analysis, specific surface area (the fitting error is provided in Table S2), particle size calculated from the specific surface area (using crystal density), and its corresponding composition characterized by ICP-MS. For reference, the composition of the commercial NMC111 was measured three times, resulting in compositions of 1.06/0.34/0.33/0.33, 1.02/0.33/0.32/0.35, and 1.07/0.34/0.34/0.33, respectively. Characterization of commercial NMC111 (size and structure) is provided in Fig. S2 and Table S1, and reproducibility data for a second batch of synthesized nanoparticles are given in Table S4

Calcination temperature	Grain size (nm)	Specific surface area (m^2^ g^−1^)	Particle size (nm)	Composition Li/Ni/Mn/Co
From flame	—	102	12	1.05/0.33/0.34/0.34
600 °C	24 ± 7	44	29	1.00/0.33/0.33/0.34
650 °C	28 ± 8	36	35	1.01/0.33/0.33/0.34
700 °C	36 ± 10	31	41	1.04/0.33/0.33/0.34
750 °C	63 ± 19	20	63	1.06/0.34/0.32/0.34
800 °C	107 ± 38	8.9	141	1.01/0.33/0.34/0.33

In terms of XRD (Fig. 2a), the samples obtained directly from the flame without post-calcination exhibit a broad XRD pattern with a relatively noisy background, suggesting multiphase which includes amorphous, rock-salt,^12^ spinel, and possibly layered phases. The peaks become sharper as the calcination temperature is increased, which can be attributed to both an increase in crystallinity and an increase in size.

Changes in the XRD spectra in the 2*θ* regions between 35°–40° (Region I) and 62°–68° (Region II) are of particular interest (Fig. 2b and c), as the layered LiNi_0.33_Mn_0.33_Co_0.33_O_2_ and the spinel phases can be well-distinguished in these regions. After calcining at 600 °C, the peaks slightly shift to larger 2*θ* values, accompanied by the emergence of the spinel phase, evident in the appearance of its (311) reflection. With an increase in calcination temperature, the peaks at around 38° and 65° exhibit an increasing tendency toward peak splitting. At 700 °C, the peak near 65° shows signs of a merged doublet peak, indicating the evolution of a layered structure. Upon calcining at 750 °C, the splitting of the (006)/(102) and (008)/(110) peaks becomes evident in both Region I and Region II, respectively, as expected for the layered rhombohedral structure. The XRD pattern of the powder calcined at 800 °C shows a single phase corresponding to a well-ordered layered structure, with distinct double peaks for the (006)/(102) and (008)/(110) reflections.

Rietveld refinement on XRD data, using the layered structure model within the *R*3̄*m* space group, is shown in Table 2. The powder calcined at 600 °C has a *c*/*a* ratio of 4.93, which is larger than the value of an ideal cubic close-packed structure (4.90),^48^ suggesting distortion of the oxygen sublattice from a face-centered cubic (FCC) arrangement along the hexagonal *c*-axis. The *c*/*a* ratio increases with the increase of calcination temperature, indicating the development of a well-organized layered structure.

**Table 2:** Rietveld refinement of as-synthesized NMC111 nanoparticles calcined at various temperatures, and commercial NMC111 as reference. The corresponding Rietveld refinement plots are provided in Fig. S5 and S2a

	Calcination temperature	Rietveld refinement				
		*a*	*c*	*c*/*a*	Li/Ni antisite defects (%)	*R*_wp_ (%)
As-synthesized nanoparticles	600 °C	2.860(1)	14.111(3)	4.93	11.4(2)	5.05
	650 °C	2.859(1)	14.144(8)	4.95	8.5(2)	4.01
	700 °C	2.858(3)	14.148(2)	4.95	7.8(1)	4.15
	750 °C	2.858(1)	14.198(1)	4.97	3.8(1)	3.33
	800 °C	2.861(0)	14.227(0)	4.97	3.5(1)	2.78
Commercial		2.859(1)	14.224(1)	4.98	3.8(1)	2.69

### Electrochemical properties

**Fig. 3:**
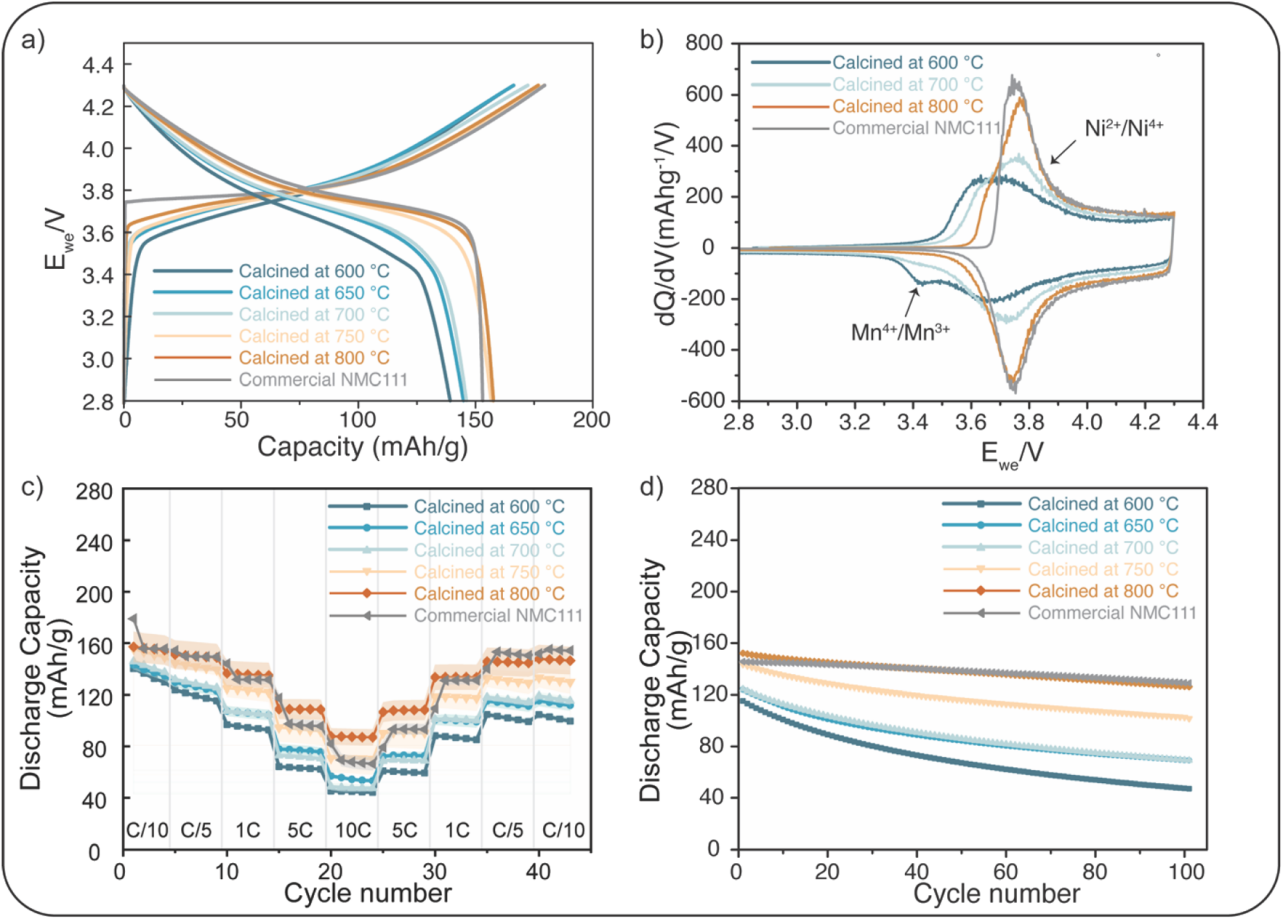
Electrochemical behavior of as-synthesized NMC111 nanoparticles. (a) Charge and discharge profiles of the 1st cycle at C/10. (b) d*Q*/d*V* plot for the 3rd cycle at a rate of C/10. (c) Rate capability test. The error bars represent the standard deviation calculated from three cells. The detailed standard deviations of the commercial NMC cells can be found in Table S7.† (d) Cycling performance at C/3 in the voltage range from 2.8 V to 4.3 V. Four formation cycles at C/10 were performed before the long-term cycling at C/3 to form a stable CEI (cathode electrolyte interphase) layer.

The charge and discharge profiles of the as-synthesized NMC111 nanoparticles cycled at a rate of C/10 are shown in Fig. 3a. The profiles of the NMC111 nanoparticles calcined at 750 °C and 800 °C align with the characteristics observed in the commercial micro-sized NMC111 particles, and they demonstrate a comparable discharge capacity to that. The initial irreversible capacity loss also decreases as the specific surface area is reduced (Fig. S8†). The d*Q*/d*V* plot of the nanoparticles calcined at 600 °C (Fig. 3b), shows that in addition to Ni^2+^/Ni^4+^ redox peaks in the region from 3.6 to 3.9 V,^49,50^ a distinct reduction peak arises between 3.3 V and 3.5 V, which is generally attributed to Mn^4+^/Mn^3+^ reduction^51,52^ and the existence of the spinel phase. Consistent with observations from XRD, with the increase of the calcination temperature, the Mn^4+^/Mn^3+^ peak gets weaker, and d*Q*/d*V* profiles corresponding to the nanoparticles calcined at 800 °C agree with commercial NMC111 with no sign of Mn^4+^/Mn^3+^ reduction.^53^ Ku *et al.*^37^ used STEM to reveal multiphase domains in the FSP-synthesized NMC particles, which is consistent with the features we observe in our d*Q*/d*V* results. When the calcination temperature reaches 900 °C, the spinel redox feature emerges again, as shown in Fig. S3d,† indicating the evolution of a spinel phase caused by significant Li loss at this temperature.

Rate capability tests of the as-synthesized NMC111 nanoparticles are performed by galvanostatic cycling involving stepwise increments in C-rate (Fig. 3c). The rate performance improves with increasing calcination temperature, as the phase purity is enhanced and surface side reactions and reconstruction are mitigated. The nanoparticles calcined at 800 °C, which exhibit a layered structure, demonstrate a good rate capability, with a specific capacity in the range of 160 mA h g^−1^ to 100 mA h g^−1^. At low currents (C/10 and C/5), the performance of the particles calcined at 750 °C and 800 °C is similar. However, at higher C-rate, particles calcined at 800 °C, although having approximately 70% larger grain size (Table 1) than those calcined at 750 °C, exhibit superior rate performance. While smaller particles typically perform better at high rates due to their shortened diffusion length,^54^ this is not observed here. We attribute the superior performance of the 800 °C particles to their smaller number of Li/Ni antisite defects compared to the 750 °C particles (Table 2), as antisite defects are tied to impede lithium diffusion.^55^ Consistent with this, GITT measurements (Fig. S7b†) reveal that the 800 °C particles exhibit higher apparent Li^+^ diffusion coefficients, and EIS measurements (Fig. S7a†) shows a smaller high-frequency semicircle compared to the 750 °C sample. We further suggest that the lower performance of electrodes with smaller particles (*i.e.*, those calcined at 750 °C) at high rates is due to limitations in electronic transport, which can be worse in the case of electrodes with many small particles with more surface area and potentially worse connectivity to the conductive carbon network.

Fig. 3d presents the capacity fade of the nanoparticles. Smaller nanoparticles exhibit larger surface areas, increasing the amount of unintended reactions occurring,^2^ like side reactions with electrolytes and transition metal dissolution, leading to a larger capacity loss (59% for nanoparticles 600 °C). The nanoparticles calcined at 800 °C, however, have only a 17% capacity fade over 100 cycles, comparable to the commercial one (11% capacity fade).

### Removal of surface impurities

It is known that atmospheric exposure leads to surface degradation of NMC111 nanoparticles.^10,39^ Moisture causes Li^+^/H^+^ exchange, resulting in the formation of surface impurities like LiOH.^56^ Some hydroxide groups can further react with CO_2_ in the air, forming carbonate groups on the surface,^40^ which can lead to electrochemical performance deterioration.^39^ To counteract this, we wash the particles with a dilute acetic acid solution. While water,^57^ ethanol^58^ and other acids like boric acid^59^ and phosphoric acid^60^ have been used to remove surface residuals on NMC particles, acetic acid benefits from its weak nature and volatility, allowing effective removal of carbonates without leaving residuals on NMC surfaces. The XRD pattern and ICP results confirm that the acid-washed particles retain their layered crystal structure, with the ratio of the transition metals remaining unchanged (Fig. S4, S6 and Table S3†).

**Fig. 4:**
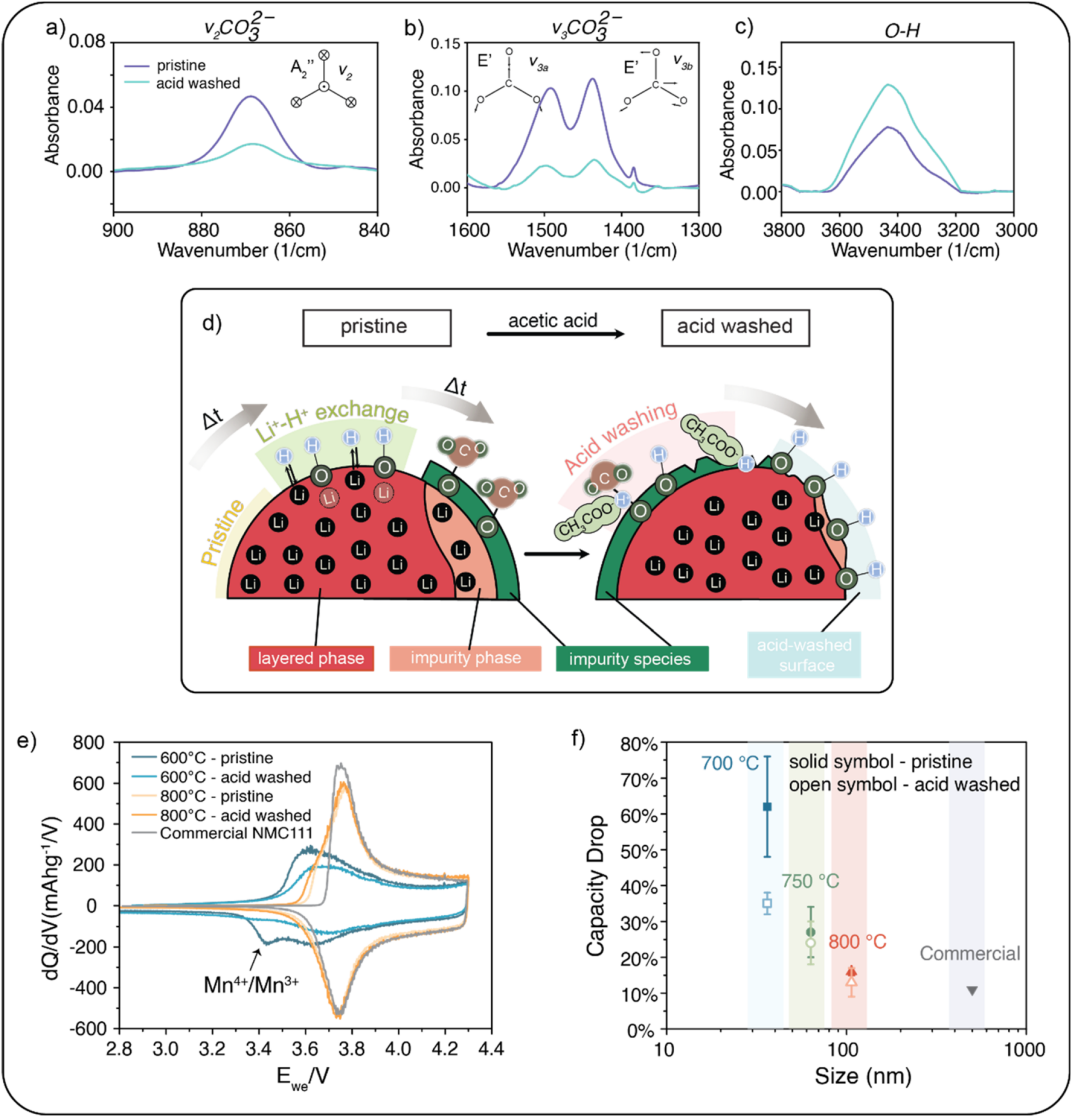
(a–c) FTIR spectra of the NMC111 nanoparticles calcined at 600 °C before and after acid washing. (d) Scheme of the acid-washing process. (e) Comparison of d*Q*/d*V* plot (at a rate of C/10) among the NMC111 nanoparticles calcined at 600 °C and 800 °C before and after washing. (f) The capacity degradation after 100 cycles at a rate of C/3 *versus* the size of the synthesized NMC111 nanoparticles w/wo acid washing. The error bars represent the standard deviation calculated from three cells.

To demonstrate the impact of the acid washing, NMC111 nanoparticles with the largest surface area (*i.e.*, the smallest nanoparticles, which are calcined at 600 °C) are examined. Fig. 4a and b shows three absorption peaks in FTIR, at 869, 1438, and 1492 cm^−1^, which can be assigned to the symmetric bending mode and two asymmetric stretching modes of carbonate,^61^ respectively. The peak at 3200–3600 cm^−1^ in Fig. 4c is attributed to the O–H stretching vibration band.^39^ After acid washing, the peaks from carbonate species diminish while those from hydroxide species increase due to the water adsorption on the etched surface. Acid washing also works similarly on the larger particles (calcined at 800 °C) as shown in Fig. S9 and ESI TGA data (Fig. S10†) further support the FTIR finding of reduced carbonate content after treatment. In d*Q*/d*V* analysis (Fig. 4e), the Mn^4+^/Mn^3+^ reduction peak shrinks, suggesting that some parts of the impurity spinel phase are also removed with the acid treatment.

Fig. 4d illustrates the process: application of mild acetic acid etches the surface of the aged particles, eliminating impurity species and portions of the spinel phase. The electrochemical properties of the washed NMC111 nanoparticles are tested, and the rate tests and long-term stability test results are provided in Fig. S11.† Comparing the capacity drop over 100 cycles at a rate of C/3 (Fig. 4f), we find that the acid-washed particles exhibit a lower capacity loss. The improvement from the acid-washing process decreases with increasing particle size (*i.e.*, higher calcination temperature).

## Conclusion

In summary, we used flame spray pyrolysis and post-calcination to achieve LiNi_1/3_Mn_1/3_Co_1/3_O_2_ nanoparticles with a layered structure and electrochemical characteristics similar to those found in commercial particles. Such particles can be used to systematically study surface coatings and treatments. While the synthesis is shown here for NMC111, we anticipate that it can be adapted to Ni-rich NMC nanoparticles, where the surface presence of Ni^3+^ renders them even more susceptible to degradation.^55^

## Author contributions

Conceptualization, X. Z., P. B., and V. W.; methodology, X. Z., K. E., F. M. S., P. B., and V. W.; investigation, X. Z., K. E., J. C., A. L.; writing—original draft, X. Z.; writing—review & editing, X. Z., P. B., K. E., F. M. S., J. C., A. L., R. S., C. R. M., W. J. S., V. W.; funding acquisition, V. W.; resources, C. R. M., W. J. S., V. W.; supervision, P. B. and V. W.

## Conflicts of interest

There are no conflicts to declare.

## Supplementary Material

RA-015-D5RA02976G-s001

## Data Availability

All the necessary data supporting the findings of this study are available in the main text and ESI†.
