# Supplementary material for: LiNi1/3Mn1/3Co1/3O2 nanoparticles produced by flame spray pyrolysis with crystallinity characteristics similar to commercial NMC particles
Source: RSC Adv. 2025 Aug 7;15(34):28075–83. doi: 10.1039/d5ra02976g (PMC12329498; doi:10.1039/d5ra02976g)
Supplement: RA-015-D5RA02976G-s001 [file RA-015-D5RA02976G-s001.pdf]

Supplementary Information (SI) for RSC Advances.
This journal is © The Royal Society of Chemistry 2025

Supporting information

$LiNi_{1/3}Mn_{1/3}Co_{1/3}O_2$ nanoparticles produced by flame spray pyrolysis with crystallinity characteristics similar to commercial NMC particles

Xueyan Zhao[1], Peter Benedek[2], Konstantin Engel[3], Florian M. Schenk[4], Jasper Clarysse[1], Ramesh Shunmugasundaram[1], Annelies Landuyt[5], Christoph R. Müller[5], Wendelin J. Stark[3], Vanessa Wood[1,z]

[1] Materials and Device Engineering Group, Institute for Electronics, Department of Information Technology and Electrical Engineering, ETH Zurich, Gloriastrasse 35, 8092 Zurich, Switzerland

[2] Department of Chemical Engineering, Stanford University, Stanford, CA 94305, United States

[3] Functional Materials Laboratory, Institute of Chemical and Bioengineering, Department of Chemistry and Applied Biosciences, ETH Zurich, Vladimir-Prelog-Weg 1, Zurich 8093, Switzerland

[4] Chemistry and Materials Design Group, Institute for Electronics, Department of Information Technology and Electrical Engineering, ETH Zurich, Gloriastrasse 35, 8092 Zurich, Switzerland

[5] Laboratory of Energy Science and Engineering, Institute of Energy Technology, Department of Mechanical and Process Engineering, ETH Zurich, Leonhardstrasse 21, Zurich 8092, Switzerland

[z] E-mail: vwood@ethz.ch

List of Figures

Figure S1 SEM image of the NMC powder calcinated at 800°C, showing that grains fusing and necking. 4

Figure S2 Characterization of commercial NMC111. a) Rietveld refinement plots of commercial NMC111 particles. b) SEM image of the commercial NMC111 (left) and the corresponding particle size distribution histogram (right). 5

Figure S3 Characterization of NMC particles calcined at 900°C: a) morphology (SEM image), b) structure information (XRD), and comparison among particles calcined at various temperatures: c) charge and discharge profiles of 1st cycle at C/10 rate. d) dQ/dV of the 3rd cycle at C/10, showing the emergence of the spinel feature at 900°C. 7

Figure S4 a) XRD patterns of the NMC powder after calcining at different temperatures from 600 to 800°C and subsequent acid washing. XRD patterns of LiNi0.5Mn1.5O4 (spinel phase, Fd–3m, ICSD-8408[2]) and $LiNi_{0.33}Mn_{0.33}Co_{0.33}O_2$ (layered phase, R-3m, ICSD-171750[3]) are shown as a reference. 8

Figure S5 Rietveld refinement plots of NMC111 nanoparticles calcined at various temperatures, a) 600°C, b) 650°C, c) 700°C, d) 750°C, and f) 800°C. 11

Figure S6 Rietveld refinement plots of NMC111 nanoparticles calcined at various temperatures and acid-washing, a) 600°C, b) 650°C, c) 700°C, d) 750°C, and f) 800°C. 12

Figure S7 Electrochemical characterization of NMC111 nanoparticles calcined at 750 °C (light brown) and 800 °C (dark brown) after one C/10 formation cycle. a) Nyquist plots from electrochemical impedance spectroscopy (1 kHz–0.1 Hz), showing a single semicircle whose diameter at 10 Hz (annotated values) reflects the combined charge-transfer and CEI resistance. b) Apparent Li^+ diffusion coefficients determined by GITT during discharge, with each marker style representing one of three independent cells cast from the same slurry to illustrate reproducibility. 13

Figure S8 Initial irreversible capacity loss as a function of NMC nanoparticle size. Particles were calcined at 600 °C (■), 650 °C (●), 700 °C (▲), 750 °C (▼), and 800 °C (◆). Error bars represent the standard deviation of three independent cells tested at C/10. 14

Figure S9 FTIR spectra of the NMC111 nanoparticles calcined at 800°C before and after acid washing. 15

Figure S10 Comparison of TGA curves for pristine and acid-washed NMC samples. Measurements were carried out on a Mettler-Toledo TGA/DSC 3+ under dry N_2 flow (150 mL min^{-1}) with a heating rate of 10 °C min^{-1}. Region I corresponds to physisorbed water loss, while Region II reflects removal of chemisorbed water (e.g., bound hydrates) and carbonates[3].

Consistent with our FTIR data, the acid-washed samples exhibit reduced weight loss in Region II compared to the untreated material...16
Figure S11 Electrochemical behavior of NMC111 nanoparticles after acid washing. **a-b)** Rate capability test. The error bar is calculated as the standard deviation of three cells **c)** Charge and discharge profiles of the 1st cycle at rate C/10. **d)** The comparison of cycling performance between the pristine and the washed particles at rate C/3 in the voltage range from 2.8 V to 4.3 V. Four formation cycles at a rate of C/10 were performed before the long-term cycling at C/3 to form a stable CEI layer. 17

List of Tables

Table S1 Characterization of commercial NMC111 secondary particles. Secondary particle diameters were determined from SEM image analysis (mean ± SD), while BET-derived diameters were calculated assuming non-porous spheres. The smaller BET diameter likely reflects contributions from sub-micron surface roughness, cracks, and internal porosity..........5
Table S2 Fit errors for the surface areas of the NMC powders after calcination at different temperatures. The errors were obtained via the density functional theory (DFT) method.........6
Table S3 Rietveld refinement results for NMC111 nanoparticles ($LiNi_{1/3}Mn_{1/3}Co_{1/3}O_2$; space group R-3m; formula weight = 96.2 g/mol per unit; Z = 3) after calcination at various temperatures and acid washing. 9
Table S4 ICP-MS-determined compositions of a second batch of NMC111 nanoparticles after calcination, demonstrating reproducibility. 9
Table S5 ICP-MS-determined compositions of NMC111 nanoparticles after calcination at various temperatures and acid washing. 10
Table S6 Final R values of Rietveld refinement for NMC111 nanoparticles ($LiNi_{1/3}Mn_{1/3}Co_{1/3}O_2$; space group R-3m; formula weight = 96.2 g/mol per unit; Z = 3) after calcination at various temperatures and commercial NMC333. 10
Table S7 Rate-performance results for the commercial NMC sample. Values are the mean ± standard deviation from three cells prepared from the same slurry. 18

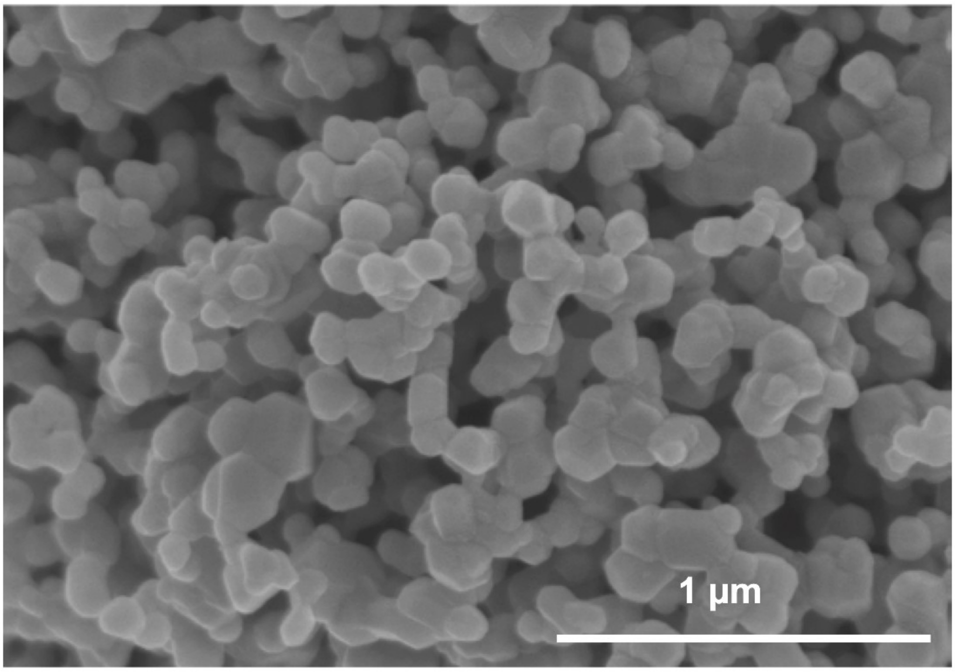

Figure S1 *SEM image of the NMC powder calcinated at 800°C, showing that grains fusing and necking.*

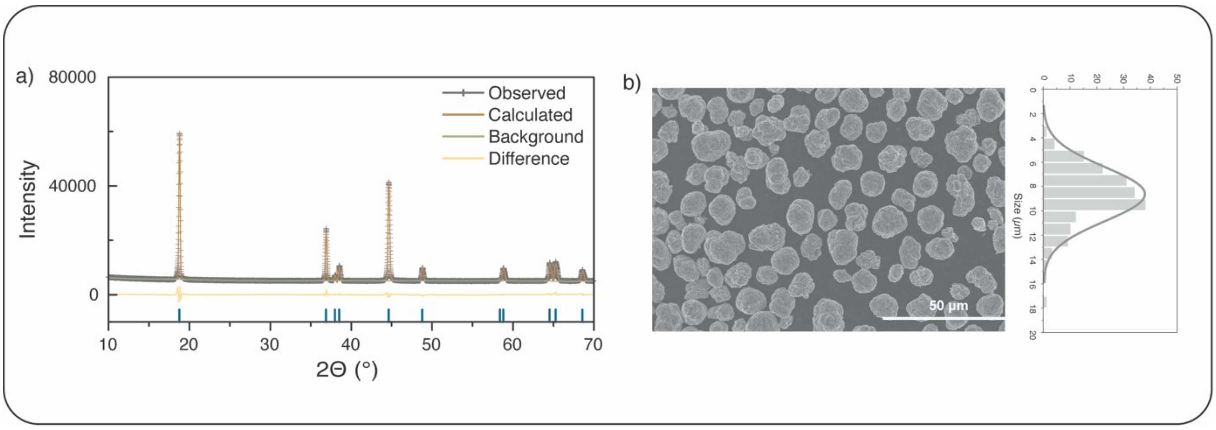

Figure S2 *Characterization of commercial NMC111. a) Rietveld refinement plots of commercial NMC111 particles. b) SEM image of the commercial NMC111 (left) and the corresponding particle size distribution histogram (right).*

Table S1 *Characterization of commercial NMC111 secondary particles. Secondary particle diameters were determined from SEM image analysis (mean ± SD), while BET-derived diameters were calculated assuming non-porous spheres. The smaller BET diameter likely reflects contributions from sub-micron surface roughness, cracks, and internal porosity.*

	Secondary particle size (µm)	Specific surface area (m^2/g)	Particle size from BET (µm)
Commercial NMC111	8.63 ±2.22	0.3246	3.9

Table S2 *Fit errors for the surface areas of the NMC powders after calcination at different temperatures. The errors were obtained via the density functional theory (DFT) method*

Calcination temperature	Fitting error
from flame	0.65%
600°C	0.64%
650°C	0.65%
700°C	0.66%
750°C	0.71%
800°C	0.67%
Commercial	0.87%

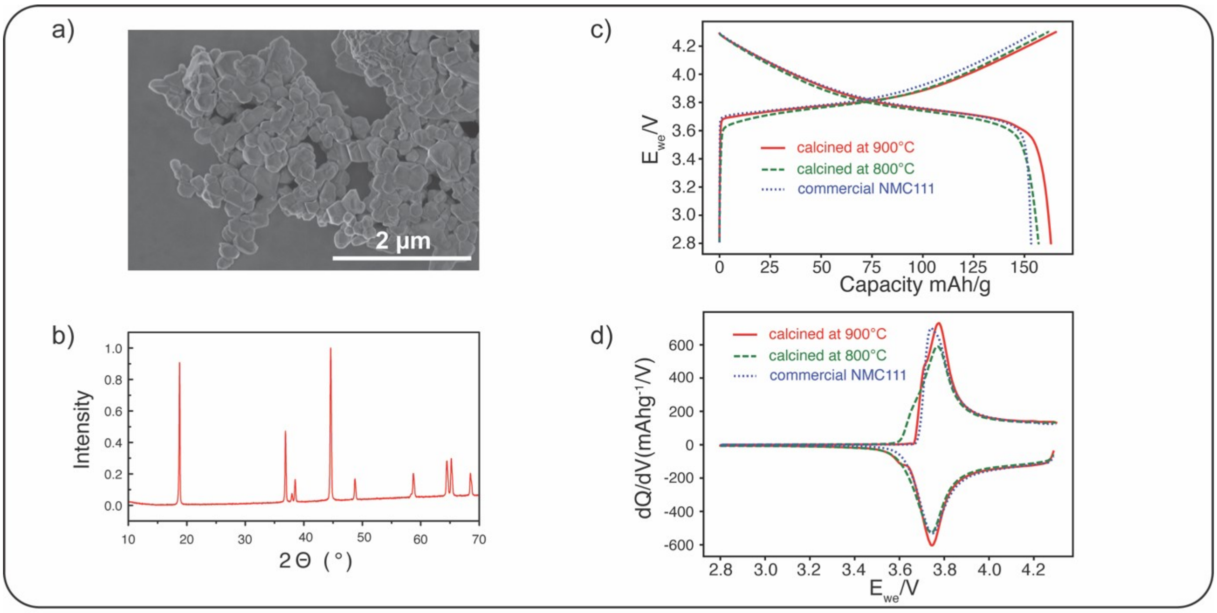

***Figure S3** Characterization of NMC particles calcined at 900°C: a) morphology (SEM image), b) structure information (XRD), and comparison among particles calcined at various temperatures: c) charge and discharge profiles of 1st cycle at C/10 rate. d) dQ/dV of the 3rd cycle at C/10, showing the emergence of the spinel feature at 900°C.*

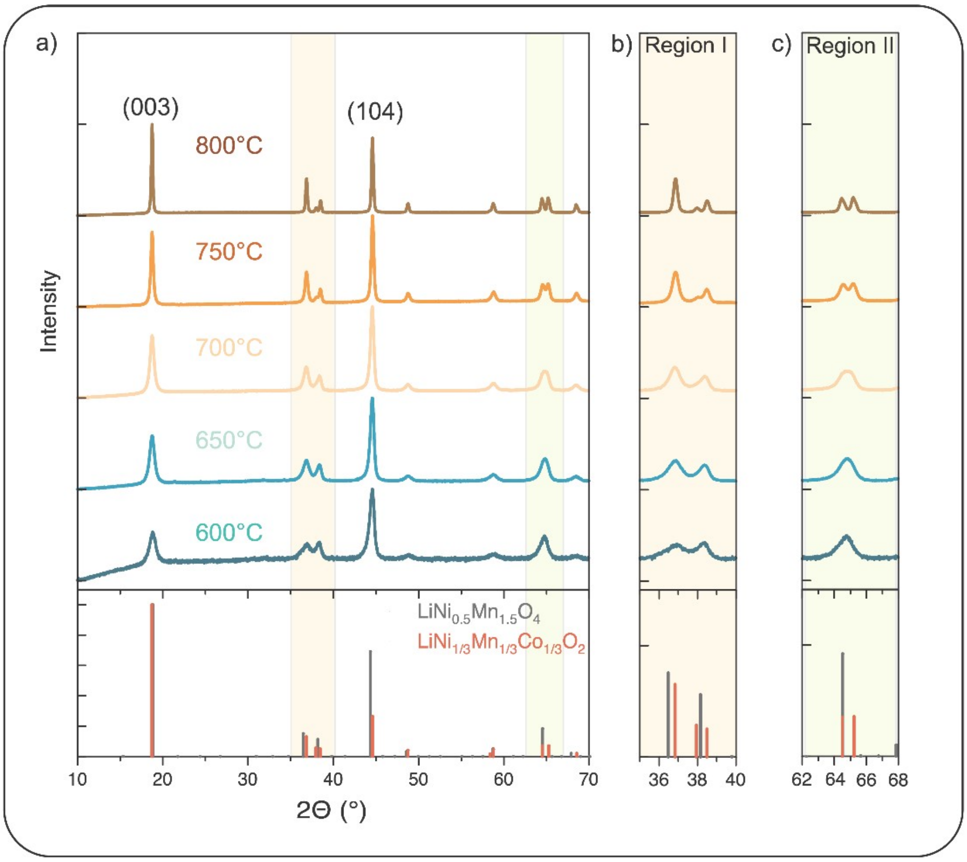

Figure S4 *a) XRD patterns of the NMC powder after calcining at different temperatures from 600 to 800°C and subsequent acid washing. XRD patterns of LiNi0.5Mn1.5O4 (spinel phase, Fd–3m, ICSD-8408*[2]*) and* $LiNi_{0.33}Mn_{0.33}Co_{0.33}O_2$ *(layered phase, R-3m, ICSD-171750*[3]*) are shown as a reference.*

Table S3 *Rietveld refinement results for NMC111 nanoparticles ($LiNi_{1/3}Mn_{1/3}Co_{1/3}O_2$; space group R-3m; formula weight = 96.2 g/mol per unit; Z = 3) after calcination at various temperatures and acid washing.*

Calcination temperature	Rietveld Refinement							
	a	c	c/a	Li/Ni antisite defects (%)	R_{wp} (%)	R_{exp} (%)	R_I (%)	Crystallite size (nm)
600°C	2.874(4)	14.218(2)	4.95	17.8(2)	4.04	3.37	4.94	15
650°C	2.865(0)	14.191(1)	4.95	13.1(1)	3.63	1.05	5.24	20
700°C	2.869(0)	14.191(1)	4.95	9.5(1)	3.74	1.07	3.10	24
750°C	2.861(0)	14.209(0)	4.97	8.9(1)	2.90	0.83	2.90	35
800°C	2.865(0)	14.276(0)	4.98	4.6(1)	4.87	4.05	1.04	67

Table S4 *ICP-MS-determined compositions of a second batch of NMC111 nanoparticles after calcination, demonstrating reproducibility.*

Calcination temperature	Composition Li/Ni/Mn/Co
from flame	1.08/0.33/0.33/0.33
600°C	1.06/0.34/0.32/0.33
700°C	1.04/0.34/0.32/0.33
750°C	1.02/0.34/0.33/0.34
800°C	1.00/0.32/0.34/0.33

Table S5 *ICP-MS-determined compositions of NMC111 nanoparticles after calcination at various temperatures and acid washing.*

Calcination temperature	Composition Li/Ni/Mn/Co
600°C	0.86/0.33/0.33/0.34
650°C	0.90/0.33/0.34/0.34
700°C	0.95/0.33/0.33/0.34
750°C	0.97/0.33/0.33/0.34
800°C	1.00/0.33/0.34/0.33

Table S6 *Final R values of Rietveld refinement for NMC111 nanoparticles ($LiNi_{1/3}Mn_{1/3}Co_{1/3}O_2$; space group R-3m; formula weight = 96.2 g/mol per unit; Z = 3) after calcination at various temperatures and commercial NMC333.*

	Calcination temperature	Rietveld Refinement		
		R_{exp} (%)	R_I (%)	Crystallite size (nm)
As-synthesized nanoparticles	600°C	2.15	5.45	17
	650°C	1.00	4.64	20
	700°C	1.17	5.58	21
	750°C	1.19	2.85	34
	800°C	1.04	1.40	66
Commercial NMC		0.25	3.24	-

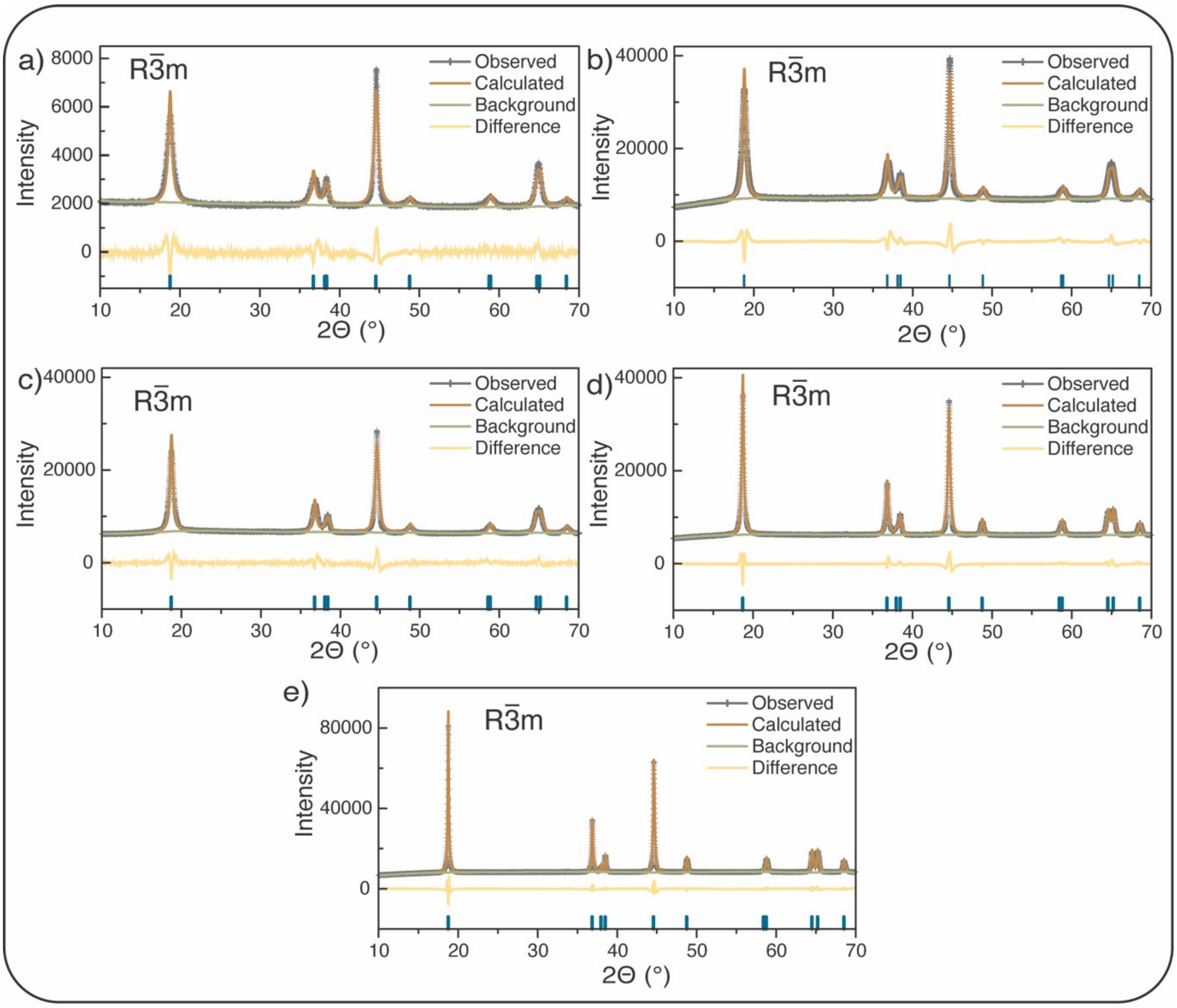

Figure S5 *Rietveld refinement plots of NMC111 nanoparticles calcined at various temperatures, a) 600°C, b) 650°C, c) 700°C, d) 750°C, and f) 800°C.*

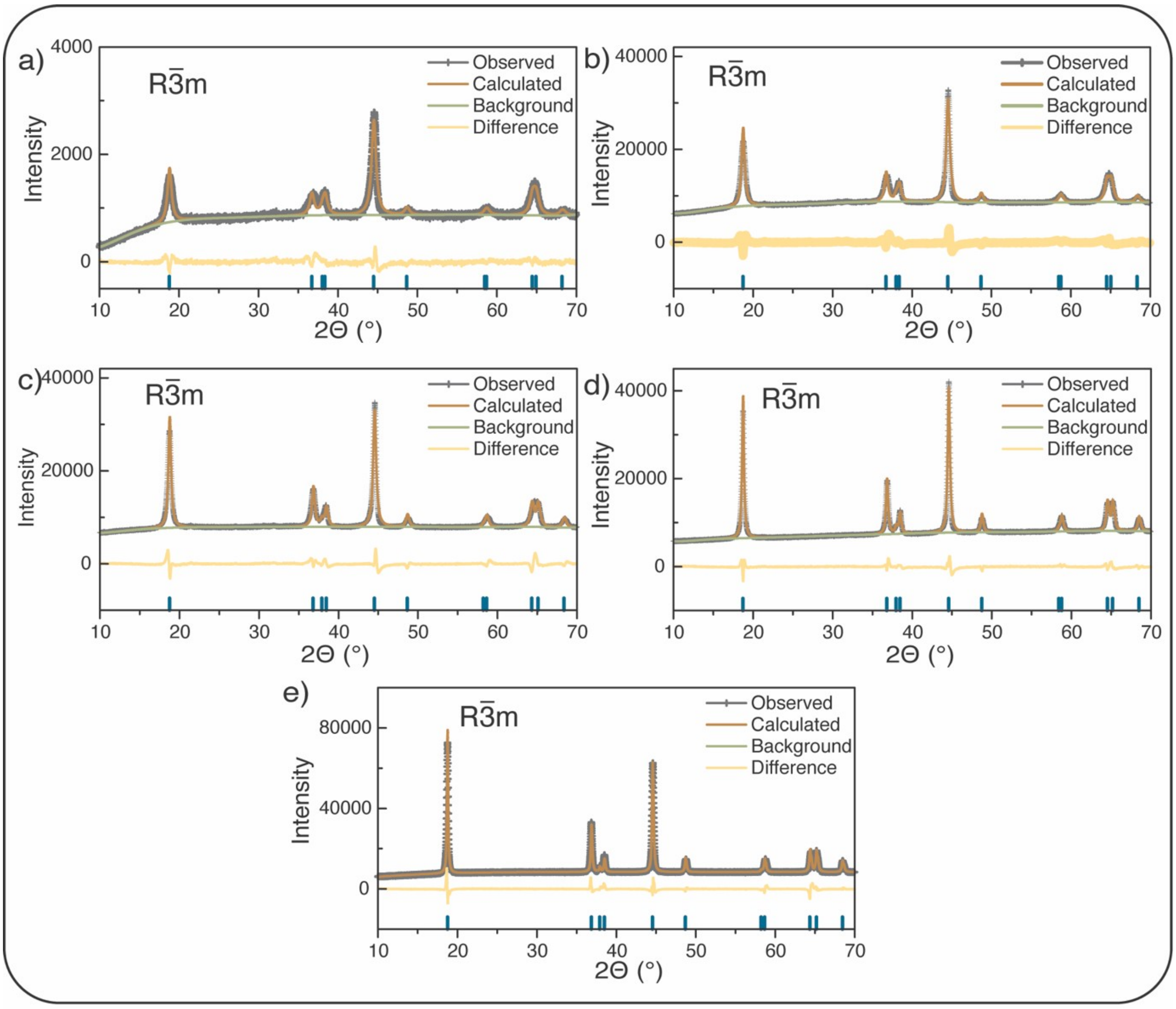

Figure S6 *Rietveld refinement plots of NMC111 nanoparticles calcined at various temperatures and acid-washing, a) 600°C, b) 650°C, c) 700°C, d) 750°C, and f) 800°C.*

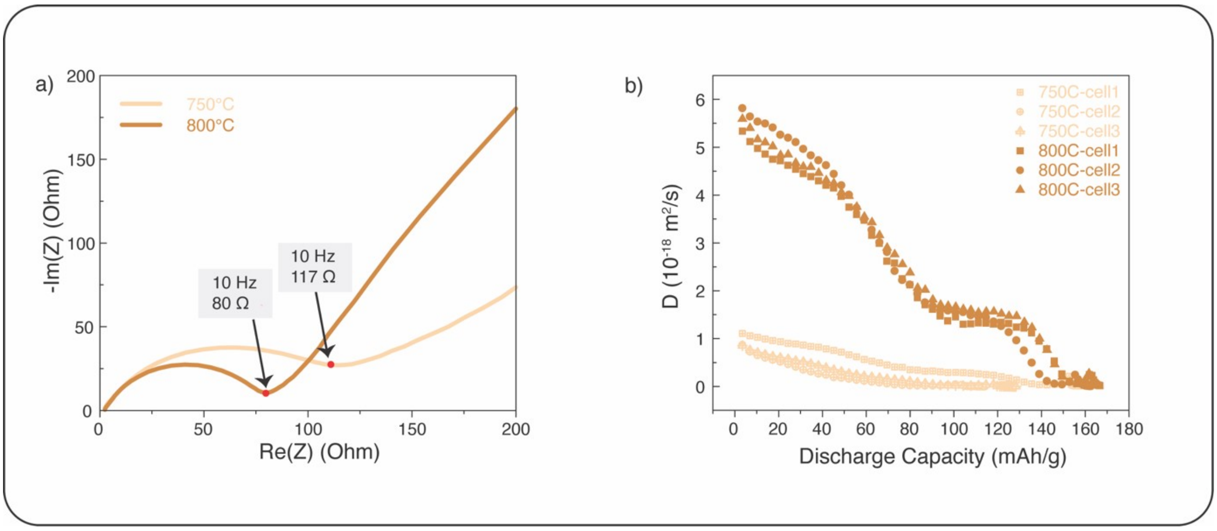

Figure S7 *Electrochemical characterization of NMC111 nanoparticles calcined at 750 °C (light brown) and 800 °C (dark brown) after one C/10 formation cycle. a) Nyquist plots from electrochemical impedance spectroscopy (1 kHz–0.1 Hz), showing a single semicircle whose diameter at 10 Hz (annotated values) reflects the combined charge-transfer and CEI resistance. b) Apparent* Li^+ *diffusion coefficients determined by GITT during discharge, with each marker style representing one of three independent cells cast from the same slurry to illustrate reproducibility.*

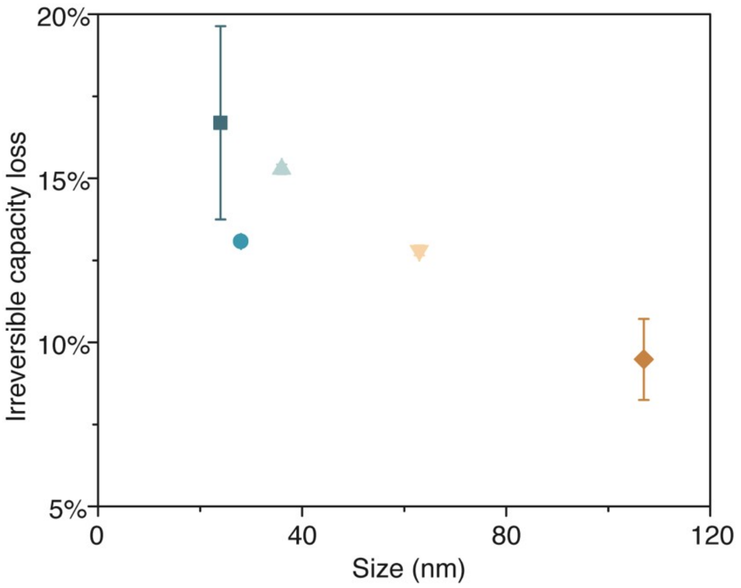

Figure S8 *Initial irreversible capacity loss as a function of NMC nanoparticle size. Particles were calcined at 600 °C (■), 650 °C (●), 700 °C (▲), 750 °C (▼), and 800 °C (◆). Error bars represent the standard deviation of three independent cells tested at C/10.*

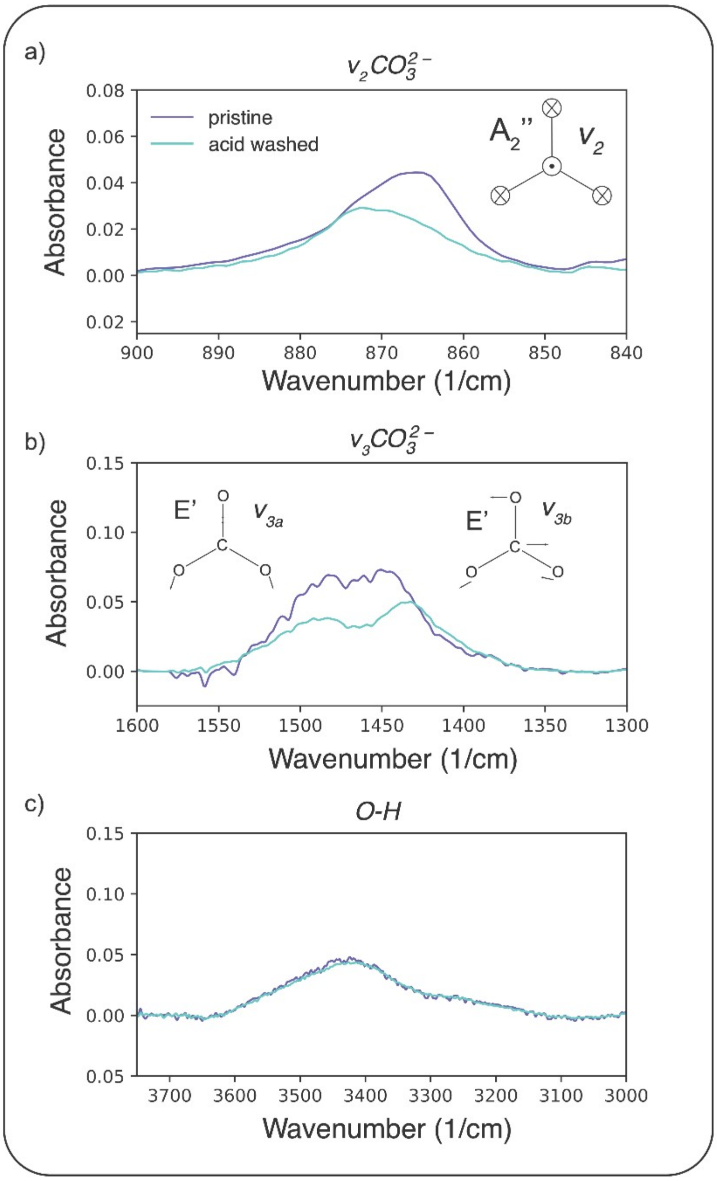

***Figure S9** FTIR spectra of the NMC111 nanoparticles calcined at 800°C before and after acid washing.*

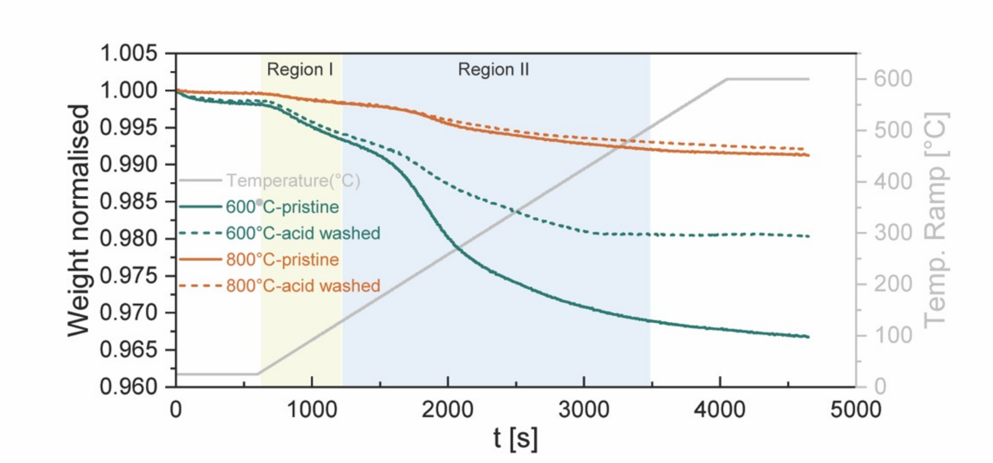

Figure S10 *Comparison of TGA curves for pristine and acid-washed NMC samples. Region I corresponds to physisorbed water loss, while Region II reflects removal of chemisorbed water (e.g., bound hydrates) and carbonates[3]. Consistent with our FTIR data, the acid-washed samples exhibit reduced weight loss in Region II compared to the untreated material.*

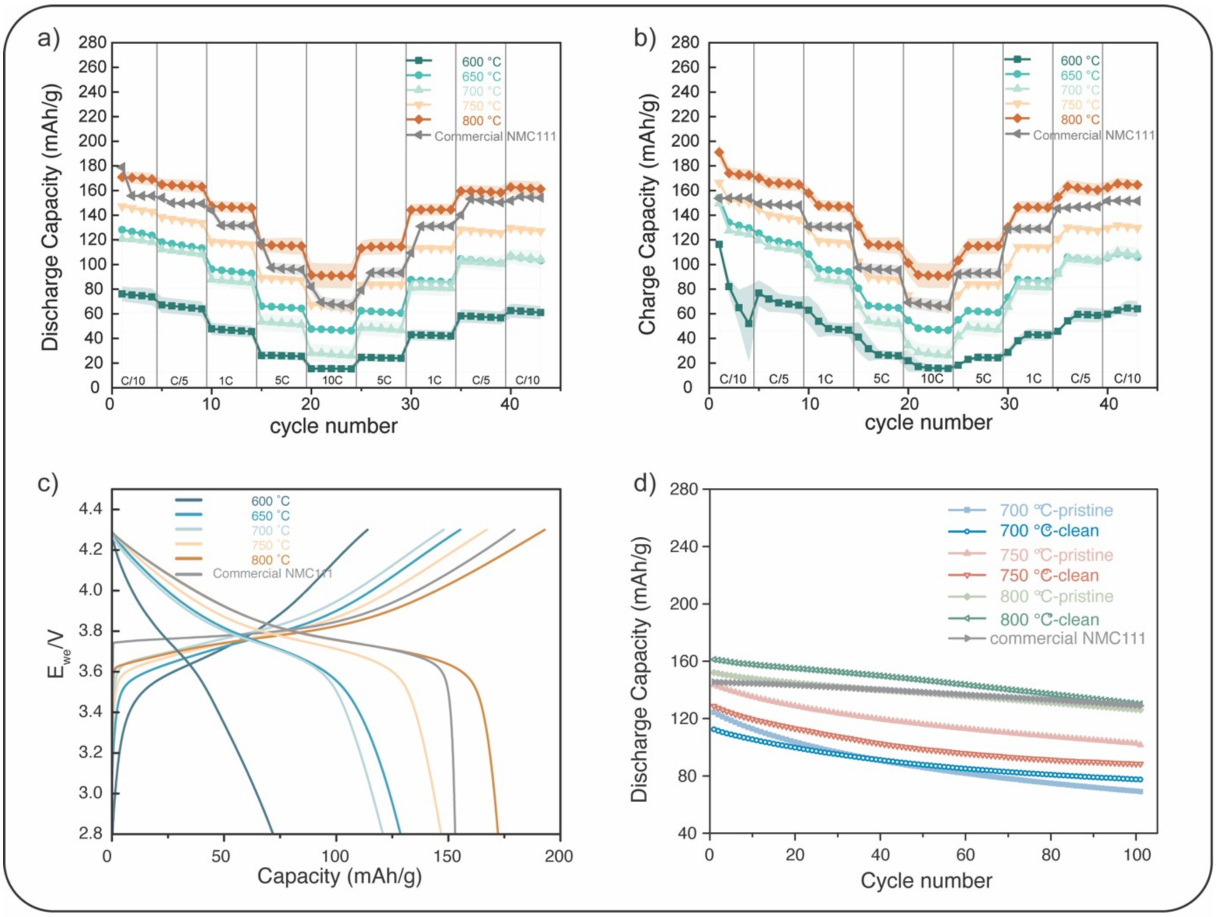

Figure S11 *Electrochemical behavior of NMC111 nanoparticles after acid washing.* ***a-b)*** *Rate capability test. The error bar is calculated as the standard deviation of three cells* ***c)*** *Charge and discharge profiles of the 1st cycle at rate C/10.* ***d)*** *The comparison of cycling performance between the pristine and the washed particles at rate C/3 in the voltage range from 2.8 V to 4.3 V. Four formation cycles at a rate of C/10 were performed before the long-term cycling at C/3 to form a stable CEI layer.*

Table S7 *Rate-performance results for the commercial NMC sample. Values are the mean ± standard deviation from three cells prepared from the same slurry.*

Rate	Discharge		Charge	
	Capacity (mAh/g)	Standard deviation (mAh/g)	Capacity (mAh/g)	Standard deviation (mAh/g)
C/10	153.73516	1.07972	178.96887	1.0731
C/10	153.9484	1.0573	155.73632	0.66513
C/10	153.85733	1.1738	155.70074	0.59816
C/10	153.70201	1.26302	155.60427	0.6083
C/5	149.2	1.33203	154.15836	0.92432
C/5	148.81043	1.49848	149.8979	1.16862
C/5	148.42072	1.74639	149.5952	1.42158
C/5	148.1419	1.91834	149.47465	1.71604
C/5	147.89011	2.14474	149.40819	2.18485
1C	130.64893	3.49923	143.92026	3.03025
1C	130.68272	3.36003	131.78294	3.30745
1C	130.59519	3.35158	131.75495	3.3189
1C	130.42633	3.36211	131.67681	3.31195
1C	130.22922	3.37232	131.55754	3.30597
5C	97.48979	4.28323	117.49653	4.03398
5C	96.58706	4.24219	97.4296	4.1367
5C	96.06403	4.29961	96.64945	4.24451
5C	95.65181	4.30647	96.15273	4.27728
5C	95.27262	4.32047	95.7527	4.29632
10C	69.15919	4.87634	81.9119	4.71072
10C	68.35954	5.02309	69.43803	4.80061
10C	67.17933	5.18161	67.89346	4.97993
10C	66.42778	5.24255	67.01013	5.05305
10C	65.86867	5.28958	66.39631	5.09891
5C	92.00355	4.44345	79.2645	4.49502
5C	92.89661	4.3233	93.28084	4.29704
5C	92.96148	4.28599	93.41478	4.25635
5C	92.8775	4.24734	93.36517	4.21891
5C	92.7194	4.21359	93.22586	4.18522
1C	128.77366	3.16257	109.48011	3.32129
1C	128.86984	3.1182	130.98259	3.03239
1C	128.93752	3.05218	131.03145	2.96358
1C	128.99141	2.98204	131.0933	2.89203
1C	129.02508	2.90841	131.14552	2.81613
C/5	145.41892	1.91586	140.24269	2.37924
C/5	146.09406	1.82554	153.16595	1.85517
C/5	146.66377	1.7027	152.28156	1.82455
C/5	147.0106	1.61609	151.23624	1.84447
C/5	147.15387	1.55327	150.30279	1.83893
C/10	151.76707	1.40718	151.88764	1.99279
C/10	151.81264	1.39242	155.15033	1.68565
C/10	151.72718	1.44858	154.5724	1.6401
C/10	151.62816	1.46705	154.12888	1.65318

Reference

(1) Chen, Z.; Wang, X.; Tian, X.; Zhong, H.; Hu, C.; Wen, J.; Peng, Y.; Xu, J.; Wu, C. Synthesis of Ordered LiNi 0.5 Mn 1.5 O 4 Nanoplates with Exposed {100} and {110} Crystal Planes and Its Electrochemical Performance for Lithium Ions Batteries. *Solid State Ion* **2019**, *333* (November 2018), 50–56.

(2) Yin, S.-C.; Rho, Y.-H.; Swainson, I.; Nazar, L. F. X-Ray / Neutron Diffraction and Electrochemical Studies of Lithium Li1-XCo1/3Ni1/3Mn1/3O2 (X=0->1). *Chemistry of Materials* **2006**, *18* (7), 1901–1910.

(3) Sicklinger, J.; Metzger, M.; Beyer, H.; Pritzl, D.; Gasteiger, H. A. Ambient Storage Derived Surface Contamination of NCM811 and NCM111: Performance Implications and Mitigation Strategies. *J Electrochem Soc* **2019**, *166* (12), A2322–A2335.
